# MEET THE END-OF-LIFE CARE EXPERTS: INSIGHTS FROM FORMER CAREGIVERS OF OLDER ADULTS LIVING WITH SERIOUS ILLNESS

**DOI:** 10.1093/geroni/igac059.839

**Published:** 2022-12-20

**Authors:** Emily Mroz, Emika Miller, Karen Moss

## Abstract

High-quality end-of-life care relies on the collective efforts of professional care providers and family caregivers; however, care practice development is rarely guided by experiential perspectives of former family caregivers (i.e., those whose care recipient has died). Former family caregivers typically carry rich, untapped insight into what works well, and what still does not, in end-of-life care. While current caregivers sometimes describe being over-burdened and unable to engage meaningfully in research, former caregivers express the desire to share insights and lessons learned to improve the experiences of future caregivers. Grounded in research methodologies which leverage insights from participants (e.g., qualitative analysis, community-based participatory research), this symposium draws on perspectives of former caregivers of seriously ill older adults to enhance end-of-life care practices, improve intervention designs, and enrich research agendas. First, Dr. Emily Mroz will describe the pathways by which recent caregiving experiences for parents living with dementia shape former caregivers’ perspectives on, and engagement in, their own end-of-life care planning. Next, Emika Miller will characterize the advance care planning perspectives of former family caregivers of African Americans who lived with dementia, highlighting areas for improvement in culturally-tailored interventions. Lastly, Jennifer Dickman Portz will present on caregivers’ use of patient portals near their care recipient’s end-of-life. Our discussant, Karen Moss, will lead us in conversation by outlining the unique value added to end-of-life care research when incorporating perspectives of former caregivers from diverse backgrounds. She will also describe future directions in caregiver-informed end-of-life interventions, practices, and policies to improve end-of-life care outcomes.

